# Combining GS-assisted GWAS and transcriptome analysis to mine candidate genes for nitrogen utilization efficiency in *Populus cathayana*

**DOI:** 10.1186/s12870-023-04202-1

**Published:** 2023-04-05

**Authors:** Xinglu Zhou, Xiaodong Xiang, Min Zhang, Demei Cao, Changjian Du, Lei Zhang, Jianjun Hu

**Affiliations:** 1 State Key Laboratory of Tree Genetics and Breeding, Key Laboratory of Tree Breeding and Cultivation of National Forestry and Grassland Administration, Research Institute of Forestry, Chinese Academy of Forestry, Beijing, 100091 China; 2 Co-Innovation Center for Sustainable Forestry in Southern China, Nanjing Forestry University, Nanjing, 210037 Jiangsu China

**Keywords:** *Populus cathayana*, GWAS, GS, RNA-seq, Nitrogen use efficiency

## Abstract

**Background:**

Forest trees such as poplar, shrub willow, et al. are essential natural resources for sustainable and renewable energy production, and their wood can reduce dependence on fossil fuels and reduce environmental pollution. However, the productivity of forest trees is often limited by the availability of nitrogen (N), improving nitrogen use efficiency (NUE) is an important way to address it. Currently, NUE genetic resources are scarce in forest tree research, and more genetic resources are urgently needed.

**Results:**

Here, we performed genome-wide association studies (GWAS) using the mixed linear model (MLM) to identify genetic loci regulating growth traits in *Populus cathayana* at two N levels, and attempted to enhance the signal strength of single nucleotide polymorphism (SNP) detection by performing genome selection (GS) assistance GWAS. The results of the two GWAS analyses identified 55 and 40 SNPs that were respectively associated with plant height (PH) and ground diameter (GD), and 92 and 69 candidate genes, including 30 overlapping genes. The prediction accuracy of the GS model (rrBLUP) for phenotype exceeds 0.9. Transcriptome analysis of 13 genotypes under two N levels showed that genes related to carbon and N metabolism, amino acid metabolism, energy metabolism, and signal transduction were differentially expressed in the xylem of *P. cathayana* under N treatment. Furthermore, we observed strong regional patterns in gene expression levels of *P. cathayana*, with significant differences between different regions. Among them, *P. cathayana* in Longquan region exhibited the highest response to N. Finally, through weighted gene co-expression network analysis (WGCNA), we identified a module closely related to the N metabolic process and eight hub genes.

**Conclusions:**

Integrating the GWAS, RNA-seq and WGCNA data, we ultimately identified four key regulatory genes (PtrNAC123, PtrNAC025, Potri.002G233100, and Potri.006G236200) involved in the wood formation process, and they may affect *P. cathayana* growth and wood formation by regulating nitrogen metabolism. This study will provide strong evidence for N regulation mechanisms, and reliable genetic resources for growth and NUE genetic improvement in poplar.

**Supplementary Information:**

The online version contains supplementary material available at 10.1186/s12870-023-04202-1.

## Background

As a rapidly growing renewable biomass and derived energy source, poplar wood can reduce dependence on fossil fuels, reduce environmental pollution, and have great economic value [1, 2]. Nitrogen (N) availability is often the main factor limiting tree productivity. N deficiency affects N uptake and assimilation, carbohydrate accumulation and distribution, and ultimately affects poplar growth and wood yield [3, 4]. Applying N fertilizers has become the main method to improve poplar wood yield in agricultural production. However, poplars have a low rate of N uptake, and excessive N fertilizer can lead to resource wastage, cause environmental pollution, and reduce economic benefits. Improving poplar NUE and identifying N use-related genes will be crucial for wood production and environmental protection.

Currently, forest tree NUE studies tend to focus on theoretical studies, with few instances of functional validation [2, 5]. The lack of genes related to N use efficiency is the main reason for this phenomenon. In the genomics era, genome-wide association studies (GWAS) with high-density marker coverage and low levels of linkage disequilibrium (LD) have developed into an efficient tool to mine key regulatory genes of target traits in populations [6–8]. However, the regulatory genes of most forest traits are often comprised of multiple genes with small effects, and effects of low-frequency alleles may not be detected by GWAS association analysis alone [8]. While increasing the sample size of the GWAS analysis population is feasible, it may not be desirable in certain scenarios. Combining GWAS with multiomics techniques will help to improve the accuracy and accuracy of candidate gene [9–11].

NUE refers to the efficiency with which plants obtain and use N [3, 12–14]. Poplar NUE research has progressed slowly, with fewer reports compared to model plants such as Arabidopsis, rice, and maize [15, 16]. Most studies have focused on N uptake efficiency by roots, with less attention given to N transport and assimilation efficiencies in xylem and phloem [17–20]. Variations in NUE can impact wood formation, which is regulated by a transcriptional regulatory network (TRN) consisting of transcription factors (TF) and genes related to the secondary cell wall (SCW) [21]. Among them, some TFs have been proven to have pleiotropic effects and participate in multiple complex metabolic processes [15, 22]. Uncovering the mechanisms underlying xylem gene responses under N treatment will provide a basis for studying the regulatory role of xylem-related genes in nitrogen metabolism.

Plants typically exhibit adaptive evolution in response to environmental changes [23, 24], including N availability, which also serves as a basis for selection in population studies. The *Populus cathayana* natural population has undergone long-term natural selection in the wild, displaying strong environmental adaptability and rich intraspecific variation. In previous research, we collected 410 genotypic *P. cathayana* germplasm resources from 34 natural populations [25, 26]. Here, we used GWAS to identify candidate genes that regulate the growth of *P. cathayana* under two N supplies and attempted to acquire genomic estimated breeding values (GEBV) by genomic selection (GS) to enhance SNPs signaling and improve GWAS mining power. In addition, we selected 13 genotypes of *P. cathayana* with high N utilization to explore the response of poplar xylem genes to N by xylem RNA-seq. Integration of GWAS and RNA-seq results provides potential genetic resources for studying NUE in poplar and strong evidence for exploring the molecular genetic basis of the response of xylem genes to N.

## Results

### Population phenotypic analysis under nitrogen treatment

Different genotype of *P. cathayana* had differences in response to N, with differences in growth indicators for most genotypes. Under two N levels, plant height (PH) and ground diameter (GD) reached significant correlation level with correlation coefficients of 0.80 and 0.85, respectively. The coefficient of variation (CV) was between 0.20–0.31. The analysis of normality using the Shapiro–Wilk test showed that the phenotype data followed a normal distribution (Figure S1). Under fertilization conditions, the average PH, average GD, and heritability remained basically unchanged. The maximum PH and maximum GD increased by 22.47% and 35.11%, respectively (Table 1). Among them, there are 159 genotypes promote PH and 191 genotypes promote GD, respectively. The ratio ranges for Ratio-PH and Ratio-GD were 1.01–2.85 and 1.01–2.62, respectively (Supplementary Table S1). Phenotypic data analysis further indicated the *P. cathayana* population has rich genetic variation and high selection potential.

**Table 1:** Statistical results of growth indices of different genotypes of *P. cathayana* in fertilization trials

Group	Trait	Mean	Range	SD	CV	H^2^
No-fertilization	PH	155.22 cm	52.00 cm-272.50 cm	44.44	0.27	0.24
	GD	12.95 mm	5.16 mm-20.93 mm	2.62	0.20	0.24
Fertilization	PH	154.33 cm	40.67 cm-333.75 cm	48.57	0.31	0.23
	GD	12.81 mm	4.99 mm-28.28 mm	2.74	0.21	0.24

### GWAS of population growth traits

**Fig. 1:**
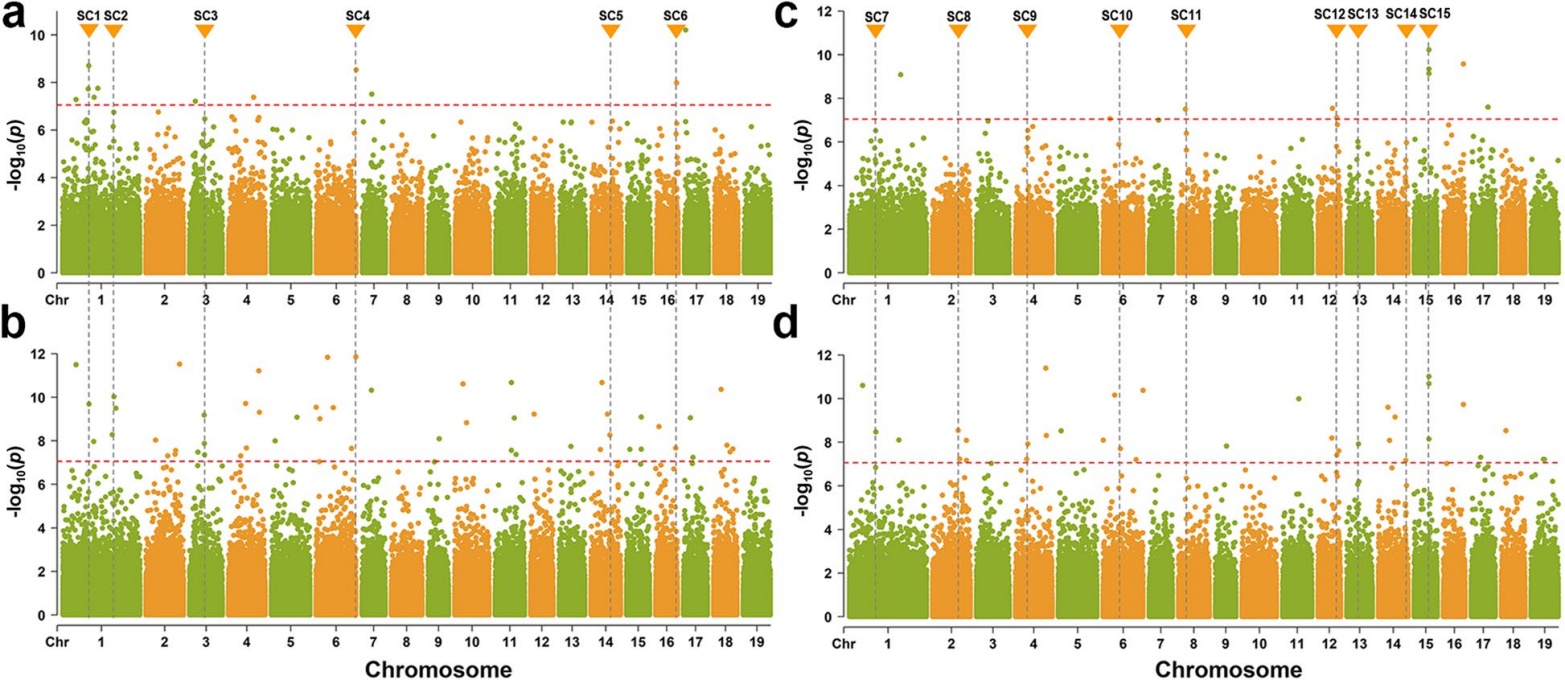
Manhattan plots of GWAS association analysis. **a** Manhattan plot of Ratio-PH GWAS correlation analysis. **b** Manhattan plot of GEBV-PH GWAS correlation analysis. **c** Manhattan plot of Ratio-GD GWAS correlation analysis. **d** Manhattan plot of GEBV-GD GWAS association analysis. *P*-values are converted to -log10 (*P-*value). SNPs above red lines passed the Bonferroni correction test, and SC1-SC15 indicate SNPs signal clusters

To identify candidate genes regulating the growth and development of *P. cathayana* under N treatment, we conducted GWAS analysis on genotypes significantly promoted by Ratio-PH and Ratio-GD (Fig. 1a, c and Fig. S2a, c). Among them, Ratio-PH was associated with 9 significant single nucleotide polymorphism (SNP) loci, and Ratio-GD was associated with 10 significant loci. We also observed multiple clusters of SNP signals in the GWAS analysis and attempted to increase the size of the GWAS population by obtaining GEBV through GS to enhance the strength of the SNP signals. GEBV analysis showed that the predictive power of PH and GD was *r*_PH_ = 0.92 and *r*_GD_ = 0.96 (Supplementary Table S1), respectively. Under this condition, the GEBV-PH trait was associated with 50 significant loci, and the GEBV-GD trait was associated with 35 significant SNPs loci (Fig. 1b, d and Fig. S2b, d).

Using the candidate intervals of 20 kb upstream and downstream of significant SNPs, a total of 92 and 69 candidate genes were identified for the PH and GD traits, respectively, with 30 candidate genes shared between the two traits. The GWAS statistics are shown in Supplementary Table S2 and S3, and the functional annotations of all genes are shown in Supplementary Table S4. The candidate genes are involved in multiple metabolic pathways, such as amino acid metabolism and biosynthesis, circadian rhythm in plants, carbon fixation in photosynthetic organisms, carbon metabolism, starch and sucrose metabolism, plant hormone signal transduction, carotenoid biosynthesis, and flavonoid biosynthesis (Supplementary Table S5). Based on gene function annotation and homology comparison, we initially identified 12 GWAS candidate genes that may respond to N treatment (Table 2).

**Table 2:** Important candidate genes and functional annotations identified in GWAS

*P. cathayana* id	*P. trichocarpa* id^a^	Trait^b^	Gene description
Pca02G020350	Potri.002G214100	GEBV-PH	Phosphoenolpyruvate carboxylase
Pca03G006430	Potri.003G072600	GEBV-PH	Alanine aminotransferase 2
Pca04G007700	NA	GEBV-GD	Transcription factor MYB20
Pca04G010300	Potri.004G118400	GEBV-PH	Myb-like DNA-binding domain
Pca04G013090	Potri.004G140900	GEBV-PH	Cytochrome P450, abscisic acid 8'-hydroxylase 4
Pca05G015040	Potri.005G164700	GEBV-GD	Cryptochrome 1 family protein
Pca06G009220	Potri.006G102600	GEBV-PH, GEBV-GD	Transcription factor bhlh140
Pca06G021060	Potri.006G236200	GEBV-PH	Auxin-responsive protein IAA2
Pca12G011040	Potri.012G126500	GEBV-GD	NAC domain-containing protein 7
Pca13G013120	Potri.013G148600	GEBV-GD	Transcription factor MYB41
Pca17G004590	Potri.017G053500	GEBV-PH	Amino acid transport and metabolism
Pca18G003350	Potri.018G036400	GEBV-GD	Nitrate regulatory gene2 protein

^a^ Gene models are annotated using v3.1 of the *P. trichocarpa* genome

^b^ GWAS traits used to localize candidate genes

### Analysis of differentially expressed genes

**Fig. 2:**
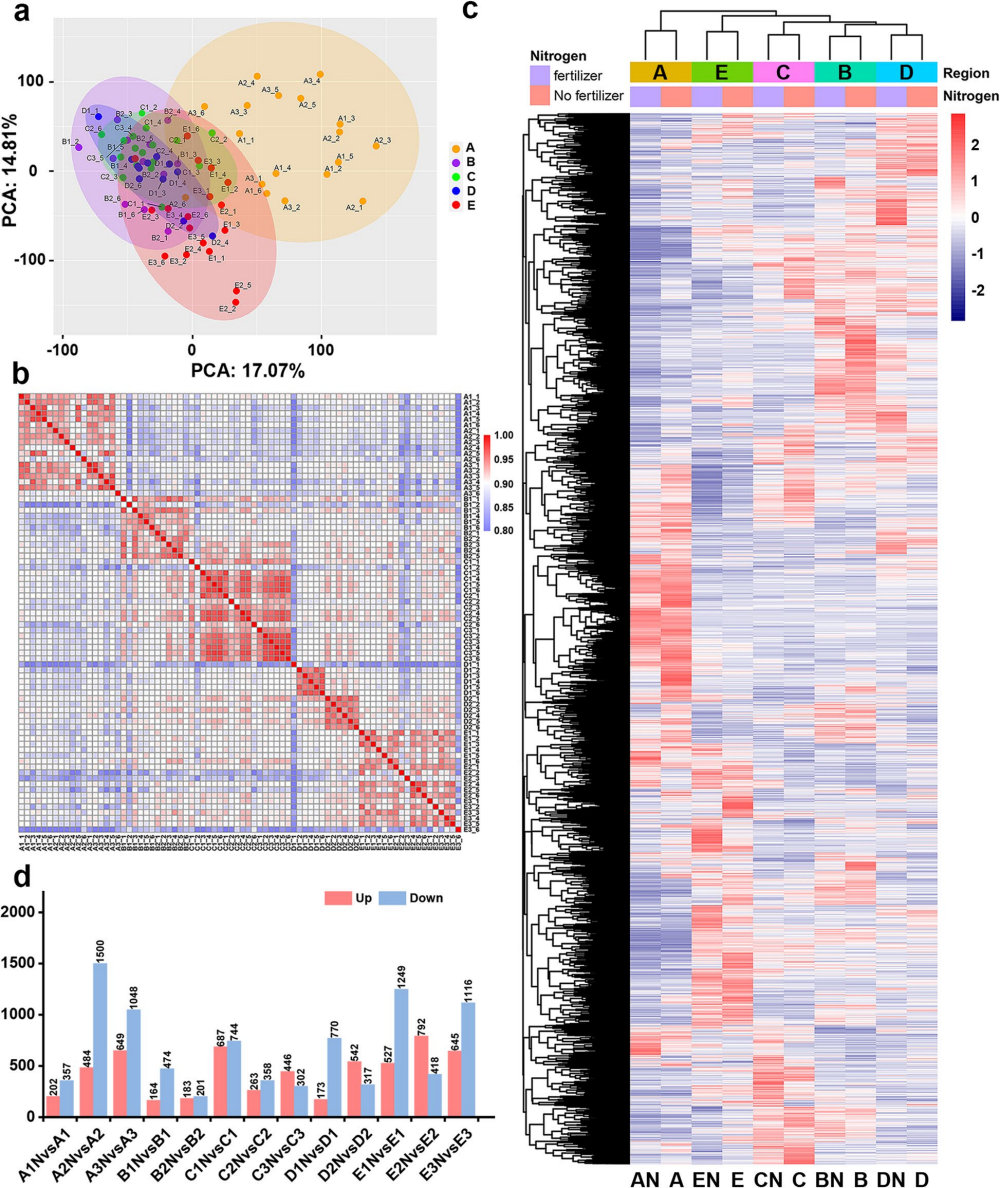
Clustering of transcriptome samples and statistical results of differentially expressed genes (DEGs). **a** Principal component analysis (PCA) of 78 samples **b** Sample correlation coefficients, with redder colors indicating higher correlation coefficients **c** Hierarchical clustering analysis of DEGs. **d** The number of upregulated and downregulated DEGs in each comparison group

**Fig. 3:**
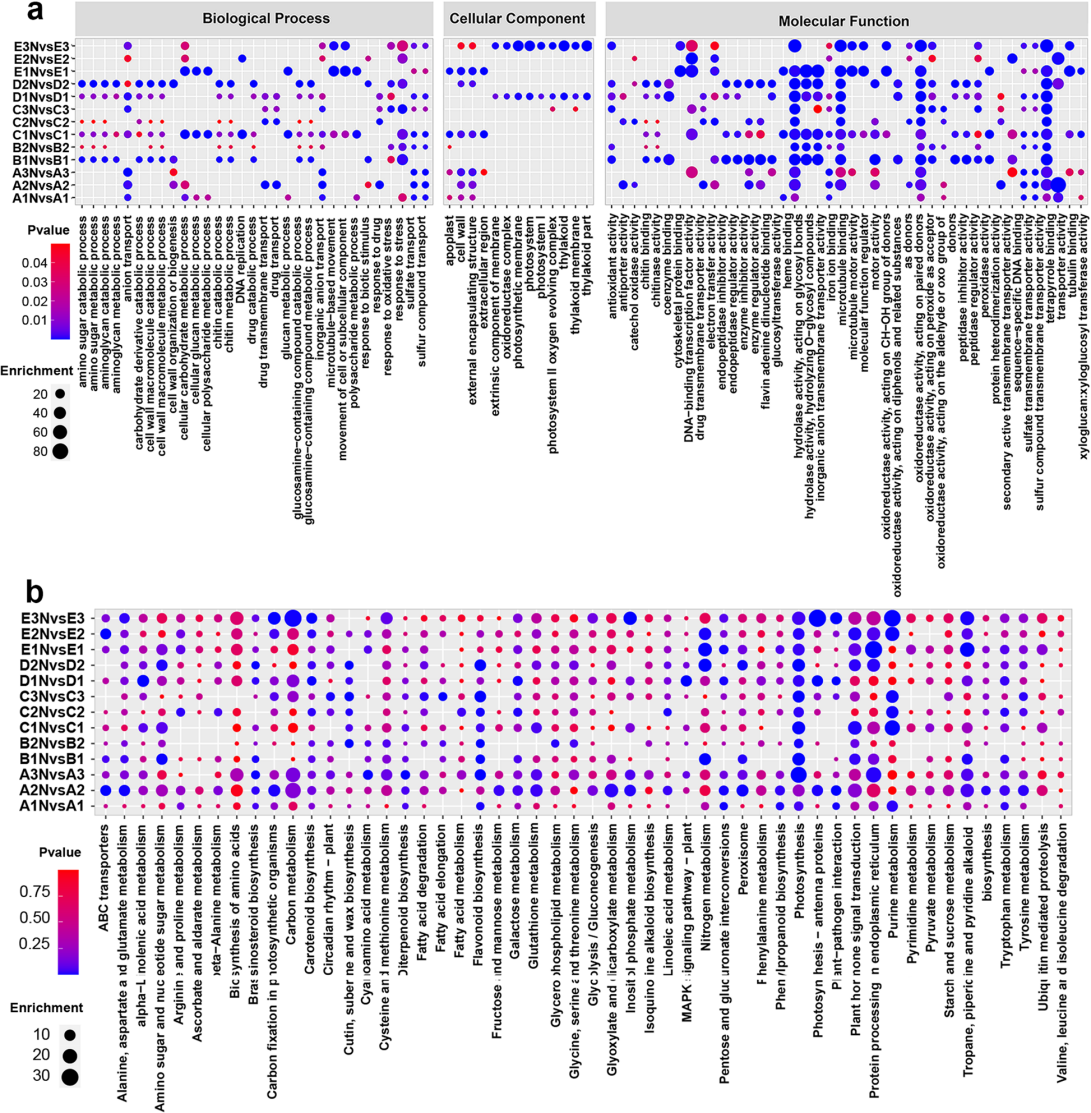
Enrichment analysis of DEGs at two N levels. **a** GO enrichment bubble diagram of DEGs. Node color and size indicate the *P*-value and the number of genes corresponding to the enrichment term, respectively. **b** Bubble diagram of KEGG enrichment of DEGs. Node color and size indicate the *P*-value and the number of genes corresponding to the enrichment term, respectively

To explore the potential N utilization-related genes involved in the growth process of *P. cathayana*, we collected the developing xylem of 13 *P. cathayana* genotypes (consisting of 5 natural populations) under two N levels and performed transcriptome analysis. Among the five natural populations we selected, the Ratio-PH and Ratio-GD populations in the Longquan area have the highest values, which are 1.45 and 1.31, respectively (Supplementary Table S6). Transcriptome analysis revealed that the expression levels of the *P. cathayana* gene exhibit strong regional pattern, and fertilization leads to differential expression of some genes. Samples from the same regional population showed high correlation, and principal component analysis (PCA) clusters into a single cluster (Fig. 2a, b, c). After quality control of the 13 sample groups (Supplementary Table S7), a total of 12,908 differentially expressed genes (DEGs) were identified (Fig. 2d). The DEGs were significantly enriched in 126 GO terms (Fig. 3a) and 119 KEGG metabolic pathways (Fig. 3b), including multiple N utilization-related metabolic pathways such as amino acid metabolism, carbon and nitrogen metabolism, energy metabolism, and signal transduction.

### Gene co-expression network construction

**Fig. 4:**
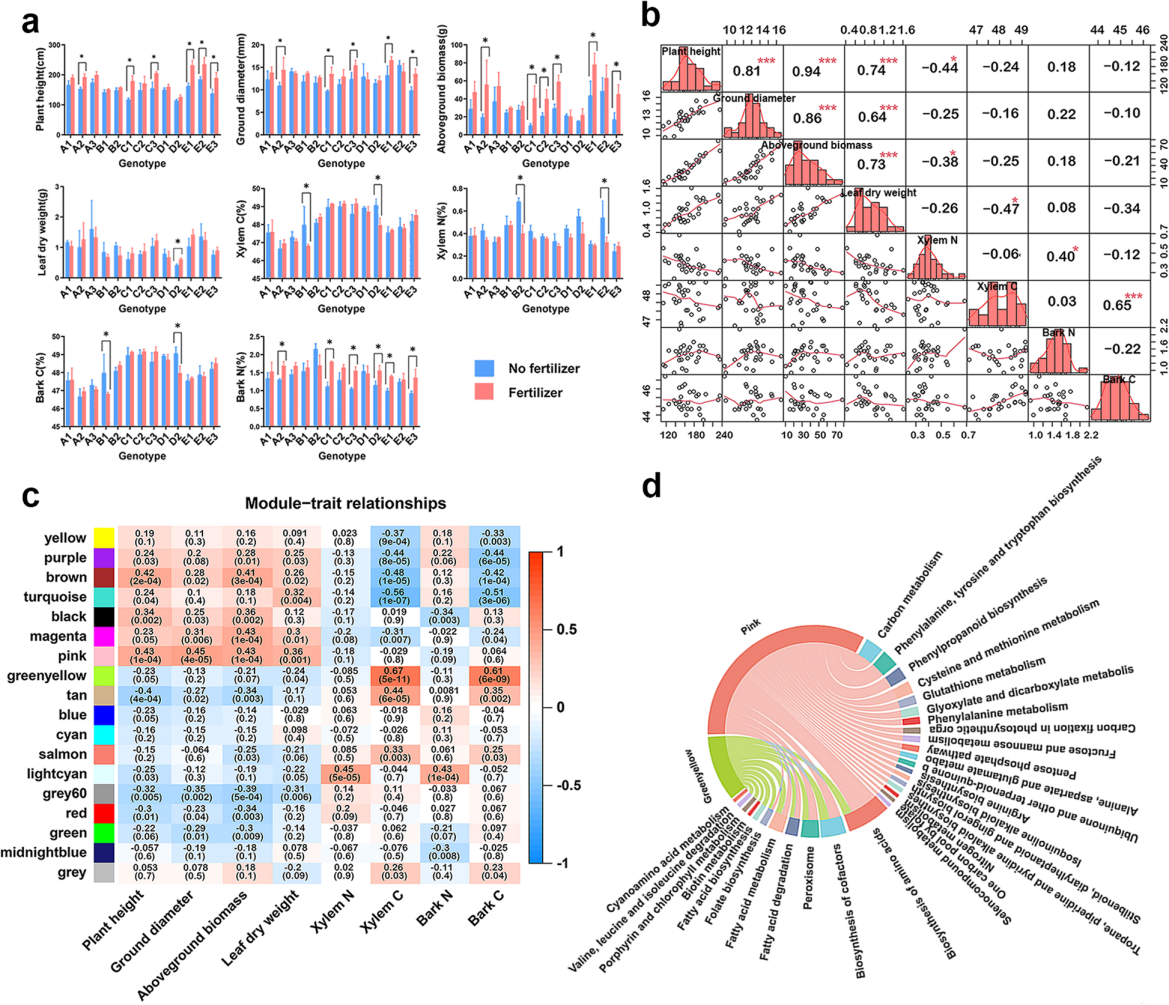
Transcriptome sample phenotypic data analysis and module association analysis. **a** Analysis of differences between the eight phenotypes data. * indicates the genotype reached a significant difference at the 0.05 level. **b** Correlations between phenotypes of all samples, * indicates a significant correlation between phenotypes at 0.05 level, *** indicates a significant correlation at 0.001 level. **c** Correlations between modules and phenotypes were based on Pearson correlation coefficients; color depth indicates the magnitude of correlation coefficients. **d** Pink, greenyellow module KEGG enrichment chord plot, the arc length corresponding to the pathway indicates the number of enriched genes; the minimum is 3 and the maximum is 32

To comprehensively analyze the response mechanism of *P. cathayana* to N, we conducted a weighted gene co-expression network analysis (WGCNA) using the FPKM value of RNA-seq gene expression in xylem samples to identify hub regulatory genes. After removing outliers (Fig. S3a), we divided all genes into 18 co-expression modules using dynamic shearing (Fig. S3b, S3c). Module-phenotype correlation analysis of the eight traits revealed that plant height, ground diameter, aboveground biomass, and leaf dry weight had similar correlations with the co-expression modules and were consistent with the interphenotype correlations (Fig. 4a, b, c). Among them, two noteworthy modules were identified: the pink module contained 799 genes (Supplementary Table S8), which were highly positively correlated with *P. cathayana* growth indicators, and the greenyellow module contained 745 genes (Supplementary Table S9), which were significantly positively correlated with xylem and bark carbon content. Furthermore, the negative correlation between xylem N and C content and other indicators indicated the close relationship between xylem carbon and nitrogen metabolism and plant growth and development.

### Module enrichment analysis and screening of hub genes

**Fig. 5:**
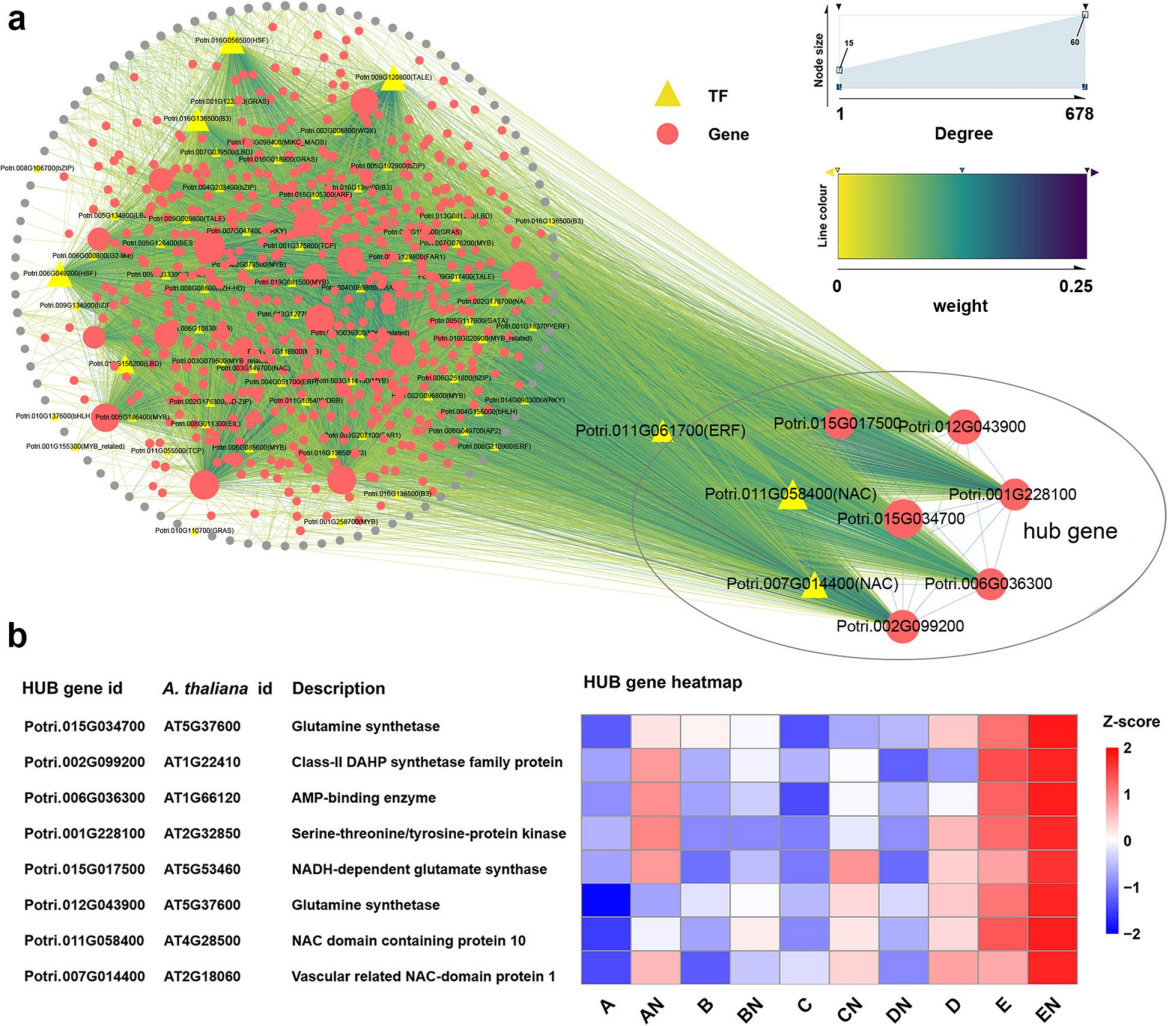
Pink module genes interactions network and hub genes analysis. **a** Visualization of the Pink module gene network, the circled parts were hub genes; the Potri.015G034700 gene regulates all genes except the gray circular parts. **b** Functional annotation of hub genes and heatmap of gene expression. Heatmap data were derived from normalized of gene expression in each region population in RNA-seq

To elucidate the biological significance of genes in the modules and identify hub genes, we conducted KEGG enrichment analysis. The pink module exhibited significantly enriched in 25 KEGG pathways (*p* < 0.05), mainly related to carbon and N metabolism, amino acid biosynthesis and metabolism of various amino acids, phenylalanine and fatty acids (Fig. 4d). Subsequently, using Cytoscape_3.7.1, we identified eight central genes from the 799 genes in the pink module, including two NAC transcription factors, Potri.011G058400 (*PtrNAC123*) and Potri.007G014400 (*PtrNAC025*) (Fig. 5a). All eight hub genes were upregulated under fertilization conditions, with four genes directly or indirectly involved in the process of N metabolism processes (Fig. 5b). The greenyellow module was enriched in 11 KEGG pathways, mainly related to the biosynthesis of cofactors, biosynthesis of amino acids, fatty acid anabolism, and catabolism (Fig. 4d). The five central genes in the module were expressed at low levels and exhibited less responsive to N, and the functional annotation information is shown in Fig. S4.

### Predicting candidate genes by GWAS and transcriptome analysis

**Fig. 6:**
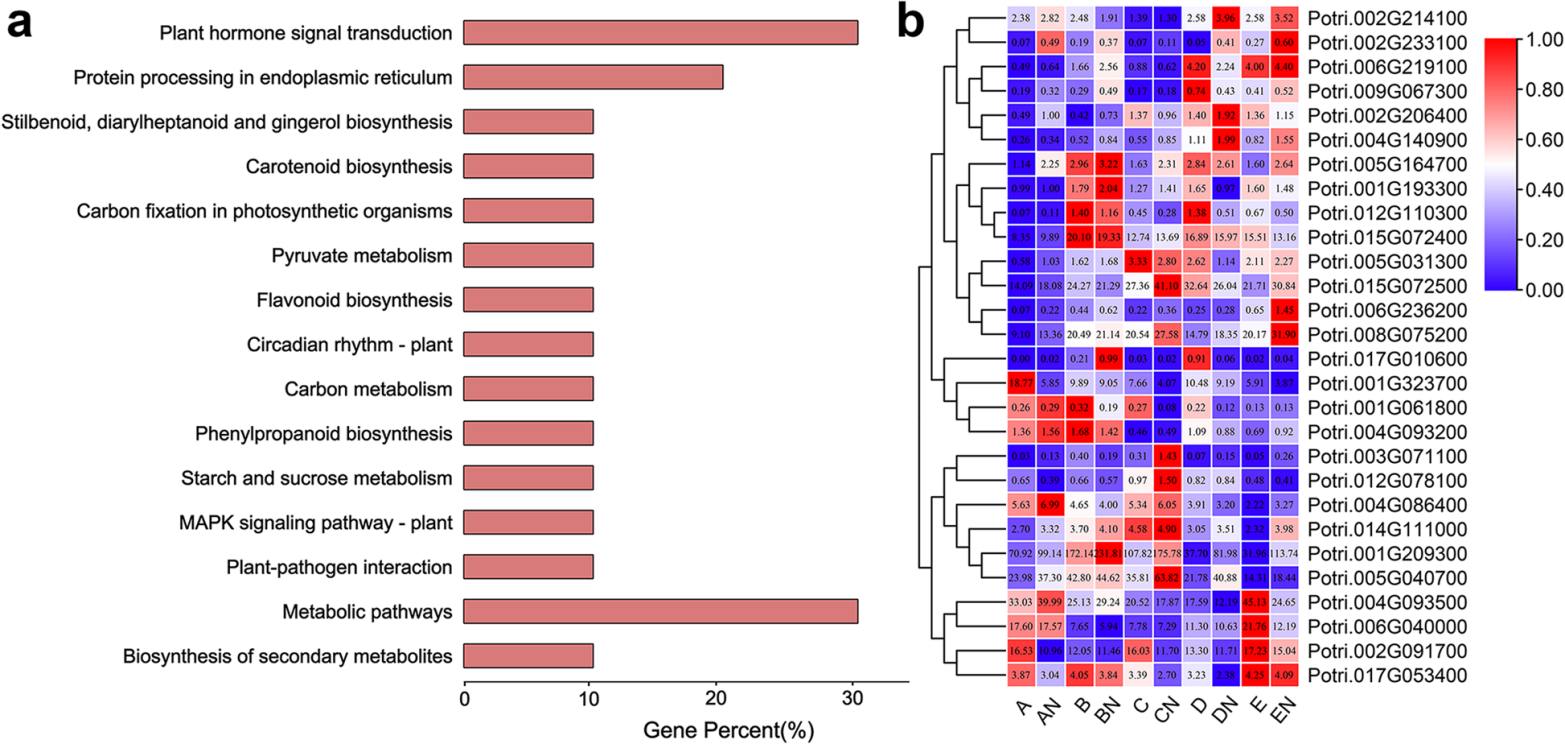
Overlapping genes KEGG enrichment analysis and expression heatmap. **a** Overlapping genes KEGG enrichment analysis. **b** Overlapping gene expression heatmap, data were derived from normalized of gene expression in each region in RNA-seq

**Fig. 7:**
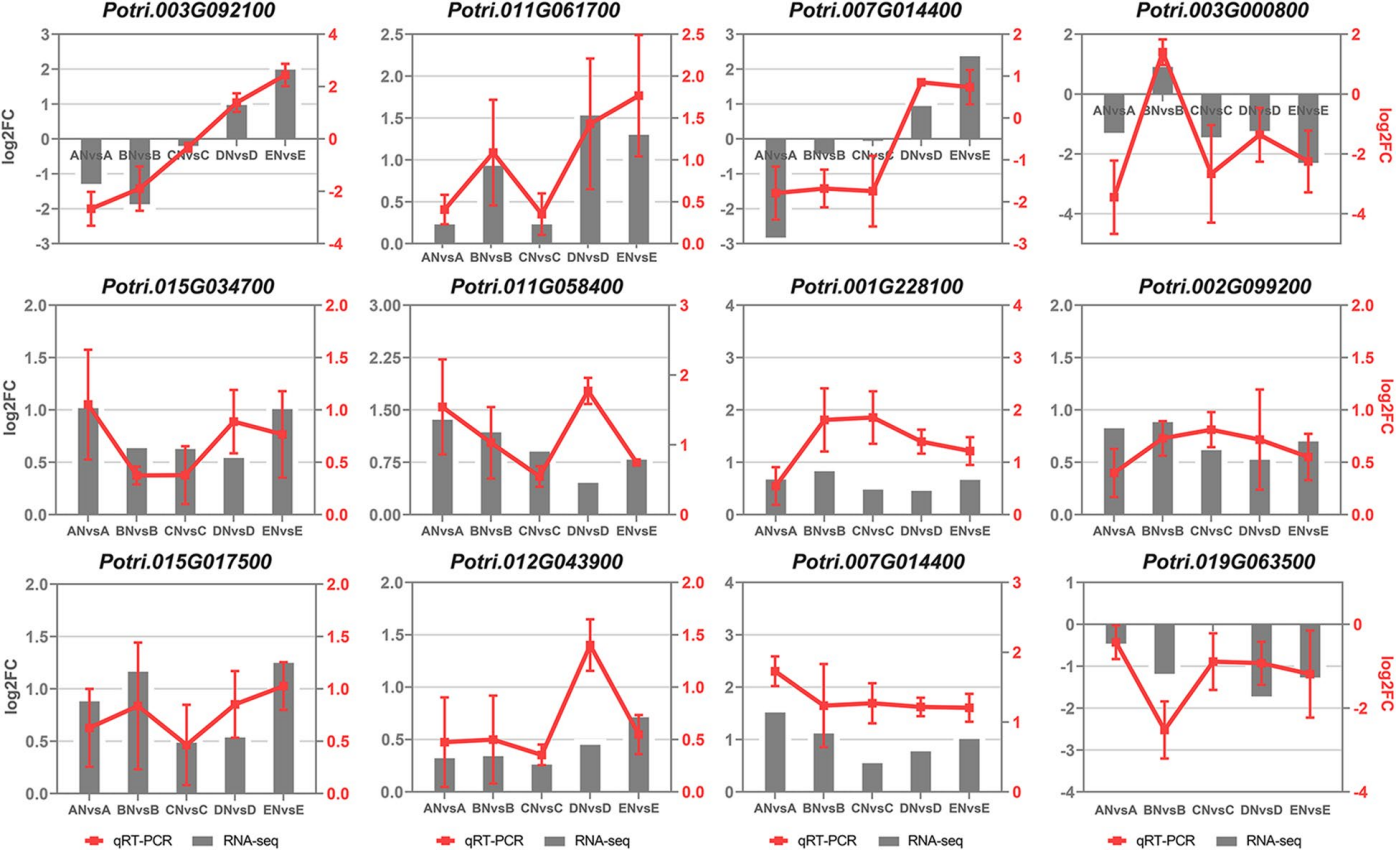
Real-time fluorescence quantification versus RNA-seq data comparison results. Three biological replicates and three technical replicates were performed for each genotype, Log2 normalized relative expression and combined with RNA-seq data to verify the accuracy of RNA-seq

To further identify the GWAS candidate genes, we overlapped them with DEGs, and obtained 28 overlapping genes (Supplementary Table S10). Subsequently, KEGG enrichment analysis was performed on these overlapping genes to understand their biological functions. The results showed that they were involved in several metabolic pathways, such as carbon metabolism, biosynthesis of amino acids, flavone and flavonol biosynthesis, plant hormone signal transduction, plant-pathogen interaction, flavonoid biosynthesis, and MAPK signaling pathway-plant (Fig. 6a). Furthermore, RNA-seq data was used to evaluate gene expression levels, which revealed that 9 genes were upregulated and 4 genes were downregulated under fertilization conditions (Fig. 6b, Table 3). Of the upregulated genes, three genes are particularly interesting: Potri.002G233100 (encoding amino acid transporter AVT1H isoform X1), Potri.004G140900 (encoding abscisic acid 8'-hydroxylase 4) and Potri.006G236200 (encoding the auxin-responsive protein IAA18-related). The quantitative real-time fluorescence PCR (qRT‒PCR) results basically showed consistency with the gene expression trends observed by RNA-seq, and the expression levels of some genes were slightly different (Fig. 7). qRT-PCR and RNA-seq results were highly correlated (*r* = 0.74; *p* < 0.001), further indicating that the transcriptomic data have high reliability.

**Table 3:** Overlapping genes with similar expression patterns and functional annotations

*P. cathayana id*	*P. trichocarpa* id^a^	Expression^b^	Trait^c^	Gene description
Pca01G017700	Potri.001G193300	up	GEBV-PH	Protein root UVB sensitive 1, Chloroplastic
Pca01G019120	Potri.001G209300	up	GEBV-PH	Basic blue protein
Pca01G030350	Potri.001G323700	down	GEBV-PH	CASP-like protein 1F3
Pca02G008700	Potri.002G091700	down	GEBV-PH	Protein NETWORKED 1D isoform X1
Pca02G021850	Potri.002G233100	up	GEBV-PH	Amino acid transporter AVT1H isoform X1
Pca03G006280	Potri.003G071100	up	GEBV-PH	17.4 kda class III heat shock protein
Pca04G013090	Potri.004G140900	up	GEBV-PH	Abscisic acid 8'-hydroxylase 4
Pca05G003430	Potri.005G040700	up	GEBV-GD	Histone H2AX
Pca06G003320	Potri.006G040000	down	GEBV-GD	F-box protein At2g26850 isoform X1
Pca06G021060	Potri.006G236200	up	GEBV-PH	Auxin-responsive protein IAA2
Pca08G006730	Potri.008G075200	up	GEBV-GD	Wound-induced protein 1
Pca14G009260	Potri.014G111000	up	GEBV-GD	Uncharacterized protein LOC7455383
Pca17G004580	Potri.017G053400	down	Ratio-GD	F-box/LRR-repeat protein 14

^a^ Gene models are annotated using v3.1 of the *P. trichocarpa* genome

^b^ Expression changes of overlapping genes

^c^ GWAS traits used to localize candidate genes

## Discussion

### Evaluation of nitrogen use of *P. cathayana* populations

Forest tree growth and development are highly dependent on N, but research on N utilization-related genes in forest tree is lacking and genetic transformation has rarely been reported [15, 27]. Therefore, there is an urgent need to identify genetic resources related to N utilization. By long-term natural selection, the *P. cathayana* population has resulted in a population with rich potential genetic resources related to NUE. However, due to the difficulties in collecting germplasm resources, no systematic evaluation of NUE in *P. cathayana* has been conducted. Different regions of *P. cathayana* exhibit varying degrees of response to N, with gene expression levels displaying a strong regional pattern. This pattern is associated with adaptive variation that has arisen from long-term natural selection in *P. cathayana.* By integrating the GEBV values for growth traits and transcriptomic data, we discovered that among the 34 regional natural populations of *P. cathayana*, the population in the Longquan area exhibited the greatest response to N. This information will contribute to the study of NUE in *P. cathayana.*

### GS-assisted GWAS enhances the signal strength of SNPs

Most traits involved in forest tree GWAS research are quantitative traits controlled by the microscopic effects of multiple genes, and require high data accuracy and population size [28, 29]. In the present study, the population size was too small under our defined significant facilitation condition, resulting in insufficient GWAS signal intensity. Therefore, it is necessary to consider methods to increase the signal intensity. Previous studies extensively reported on GWAS-assisted GS [30–35]. On this basis, Spindel's study [36, 37] confirmed the feasibility of combining the GWAS model with ridge regression best linear unbiased prediction (rrBLUP) to improve the accuracy of the GS model. Therefore, we attempted to extend GWAS population by using GS to improve the detection ability of SNPs. Our results suggest that this is a feasible approach, and the combination of the two can greatly reduce data and false positives of candidate SNPs.

### Analysis of N metabolism-related pathways

**Fig. 8:**
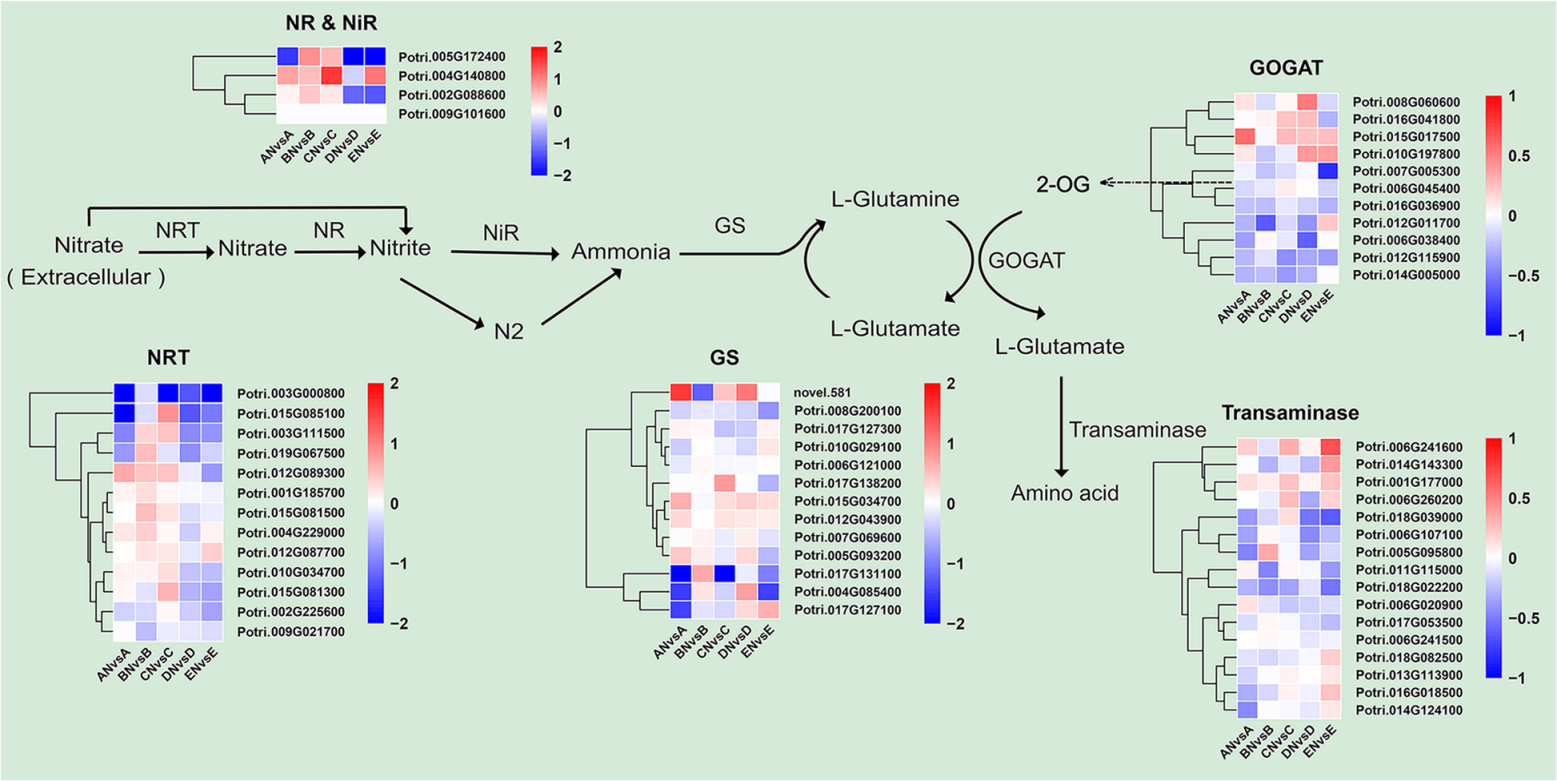
Expression changes in various enzyme-related genes during N metabolism at two N levels. NRT: nitrate transporter; NR: nitrate reductase; NiR: nitrite reductase; GS: glutamine synthetase; GOGAT: glutamate synthase; effectively expressed genes (FPKM value > 1) associated with enzymes in N utilization were screened based on gene annotation information, and heatmaps were drawn based on the difference multiplicity Log_2_FC

Carbon and N mutually regulate each other and are crucial for changing plant development and growth [38, 39]. Elucidating the expression patterns and major regulatory networks of N utilization-related genes under N treatment is of great significance for improving NUE and plant growth and development. Here, we extracted DEGs involved in amino acid carbon metabolism, nitrogen metabolism, and biosynthesis of three closely related metabolic pathways. To facilitate presentation, we plotted heatmaps using the average gene expression in each region at two N levels. Most of the DEGs associated with nitrogen metabolism and amino acid biosynthesis were upregulated under N treatment (Fig. S5). Previous studies have shown that gene expression levels [5, 40, 41] and enzyme activities associated with nitrogen metabolism differ significantly at different N levels [27, 42, 43]. We focused on examining the differences gene expression involved in major pathways of N metabolism under fertilization conditions (Fig. 8). Three hub genes (Potri.015G034700, Potri.012G043900, and Potri.015G017500) identified by WGCNA played crucial roles in this process. Based on the protein interaction network of N utilization-related genes that we constructed, we identified the important regulatory role of Potri.012G043900 (Fig. S6). This gene has catalytic activity and is involved in processes such as nitrate reduction, N compound metabolism, and glutamine biosynthesis.

### Integrating transcriptome data and GWAS to predict candidate genes

NUE affects the wood yield, and NUE regulatory genes have been shown to have multiple effects [15, 16, 22, 44, 45]. In order to explore potential NUE-related genes in the process of wood formation, we identified a WGCNA co-expression module closely related to nitrogen metabolism based on transcriptome data. The hub genes of this module include four genes directly or indirectly involved in nitrogen metabolism and two NAC transcription factors (*PtrNAC025* and *PtrNAC12*). Previously, Chen et al. [5] constructed a four-layer transcriptional regulatory network (TRN) for poplar wood formation. This network, with *PtrSND1* as the top-level regulatory factor and *PtrMYB74* and *PtrMYB21* as second-level regulatory factors, regulate the expression of 17 target genes and their downstream genes involved in secondary cell wall biosynthesis during wood formation [21, 46, 47]. One of the candidate genes we identified, *PtrNAC025* (also known as *VND6-C1*), has strong interactions with the *PtrSND1* family of regulators at the top layer of this TRN [48, 49]. The candidate gene *PtrNAC123* is located at the third layer of this TRN, and its regulated downstream gene *PtrCCoAOMT1* (Potri.009G099800) has been reported to be highly responsive to N [50], while the *PtrCCoAOMT1* gene is also located in the module we identified regulated by two candidate NAC genes.

**Fig. 9:**
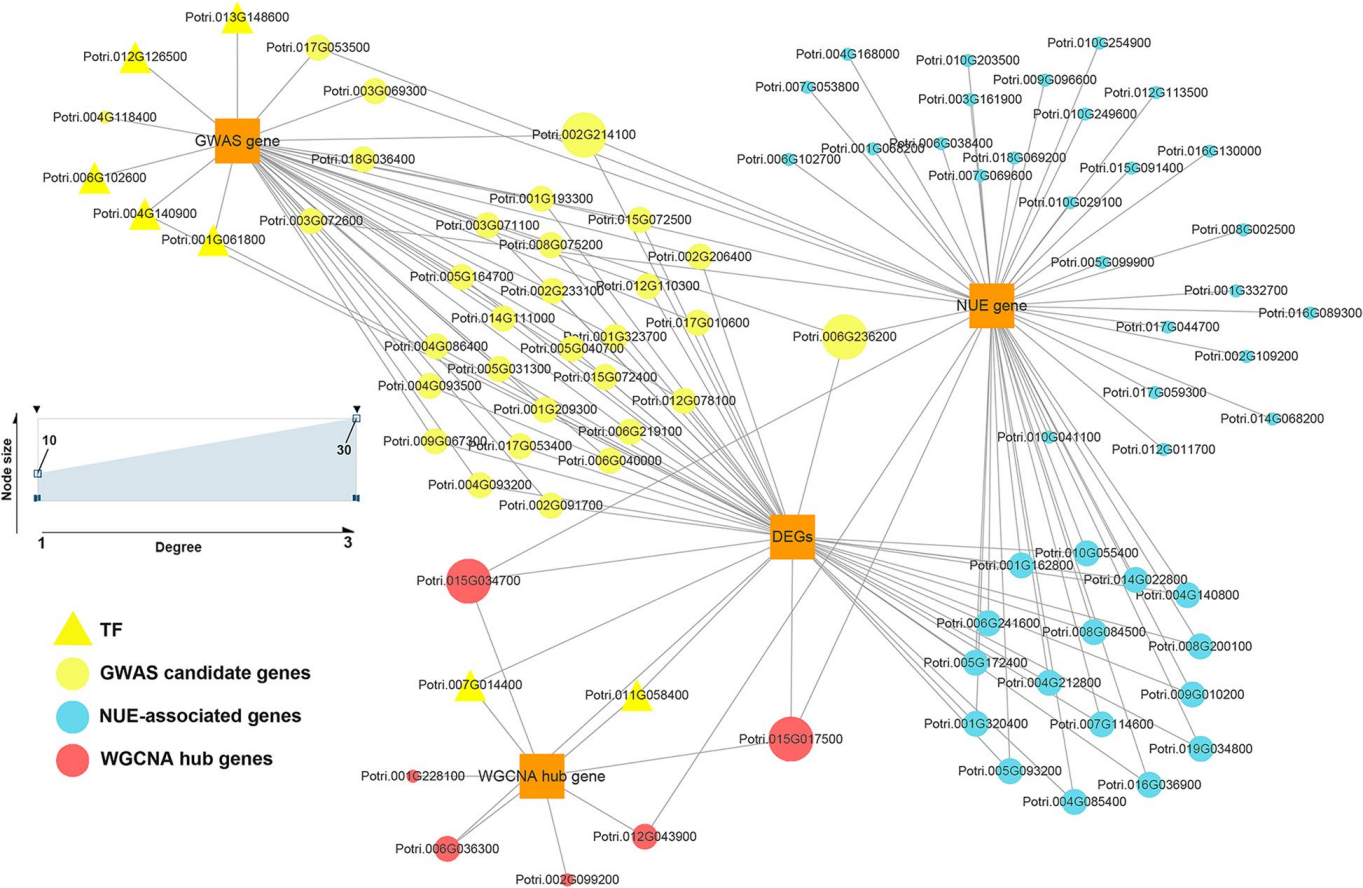
GWAS and transcriptome data network graph. The size of the dots represents the degree of gene association. The WGCNA core genes are the 8 core genes identified in the pink module, and the NUE-related genes are important genes in the nitrogen metabolism, carbon metabolism, and amino acid biosynthesis pathways

Integrating GWAS and multi-omics data has improved the accuracy and precision of candidate gene selection to some extent, and has been widely used in maize, soybean and poplar [10, 51, 52]. In this study, two GWAS candidate genes, Potri.002G233100 and Potri.006G236200*,* were identified by integrating GWAS and transcriptome data (Fig. 9). Potri.002G233100 encodes amino acid transporter family protein, and its homologous gene in *Arabidopsis thaliana*, AT5G16740 is closely associated with the transmembrane amino acid transport [53]. Potri.006G236200 encodes auxin-responsive protein IAA18-related, and its homologous gene in *Arabidopsis thaliana*, AT3G16500 encodes phytochrome-associated protein 1, which may regulate the expression of genes associated with the growth hormone response [54]*.*

## Conclusion

Here, we presented the first genome-wide study on the response of *P. cathayana* population to N and clarify the differences in N response among different populations of *P. cathayana*, with the Longquan area showing the greatest response. Differences in N utilization efficiency affect the expression levels of NUE-related genes in the xylem, thereby influencing carbon metabolism and fixation, and ultimately impacting the growth and development of poplar trees. Based on this, by integrating the GWAS, GS and xylem transcriptomic data we identified the important regulatory role of Potri.012G043900 in nitrogen metabolism, and we preliminarily suggested that *PtrNAC123*, *PtrNAC025*, Potri.002G233100, and Potri.006G236200 may affect poplar growth by regulating nitrogen metabolism levels in addition to their known functions. This study provides new genetic resources and strong evidence to explore the molecular genetic basis of NUE in forest trees, and attempts to validate the functions of these genes in subsequent research.

## Materials and methods

### Plant material and experimental design

The *P. cathayana* population included 408 genotypes from natural populations in 34 different regions of China. In early April 2021, stem cuttings were used for clonal propagation in the greenhouse, with 30 plants of each genotype. After one month of growth, they were transplanted to the Yuquan Mountain Nursery of the Chinese Academy of Forestry. They were used for field fertilization experiments in a completely randomized block design, with a total of four blocks, two N-applied and two non-N-applied. Each block included three plants of each genotype, with a spacing of 30 cm × 50 cm. N-applied blocks were fertilized four times with 4 g/plant/15 days (CON2H4, N content ≥ 46.0%), and non-N-applied blocks were left untreated. The test plots were brown soils with 1.275 g/kg total N, 0.797 g/kg total P and 16.0 g/kg total K.

### Population phenotype data determination and analysis

After growth ceased in October, the PH and GD of each tree were measured, and the phenotypic data were tested for normality using SPSS 20.0 software. The mean (Mean), standard deviation (SD), coefficient of variation (CV) and heritability (H^2^) of PH and GD were calculated for each genotype under fertilized and unfertilized conditions. Based on the trait measurements, we calculated the trait ratios of PH and GD at two N levels for GWAS analysis. To identify phenotype-associated SNPs, we selected significantly promoted genotypes for GWAS analysis. Among them, there were 159 and 191 genotypes for Ratios-PH and Ratios-GD, respectively.

### SNPs quality control and GWAS analysis

Based on the whole-genome resequenced SNPs data, we obtained a total of 575,472 SNPs data after using the quality control of Plink 1.9 software (minor allele frequency > 0.05, missing genotype < 0.05 and r^2^ > 0.20). To reduce false positives of significant SNPs in GWAS association analysis, Q matrix and K matrix were controlled in GEMMA (0.98.3) software [55] and mixed linear model (MLM). Using GEMMA (0.98.3) software to calculate Kinship (K matrix), GTAC software to calculate PCA (Q matrix). Manhattan and Q-Q plots were drawn using the package "CMplot" package in R. The significance threshold for SNP markers was corrected by Bonferroni P = 0.05/n, where n indicates the number of valid independent SNPs. The GWAS model was Y = Wα + xβ + u + e, where Y is an n-vector of quantitative traits (or binary disease labels) for n individuals; W is a n × c matrix of covariates (fixed effects), it should contain a first column with an intercept of all 1 s; α is a c-vector of the corresponding coefficients including the intercept; x is an n-vector of marker genotypes; β is the effect size of the marker; u is an n-vector of random effects; and e is an n-vector of errors.

### GS-assisted GWAS analysis

In the GWAS analysis, we found the presence of multiple signaling clusters of SNPs, which may have been false negatives after Bonferroni correction. To further identify phenotype-associated SNPs, we attempted to use the GEBV obtained from GS by the rrBLUP method and to use them for GWAS to expand the breeding population and enhance the signal strength of SNPs. The quality control conditions of genotype data for GS analysis are the same as those for GWAS analysis. First, we used the significantly promoted genotypes as the reference population, and the remaining genotypes as the validation population. Based on the ratios of the PH and GD traits at two N levels in the reference population, genome selection analysis was performed using the R package "rrBLUP" to obtain the GEBV value of each genotype. The predictive ability (*r*_gy_) was estimated as the correlation between the observed and the GEBV (r (y, GEBV)) [56]. The GS model in the R package "rrBLUP" was Y = Xb + Za + e, where Y is the phenotypic measure of the trait being analyzed; X and Z are incidence matrices for the vectors for parameters b and a, respectively; b is a vector of fixed block effects; a is a vector of random additive effects, and e is the random residual effect.

### Identification of significant SNPs and candidate genes

Here, to reduce the false positives of SNPs loci, we defined two categories of loci significantly associated with phenotypes as the final significant SNPs loci. The first category of SNPs are the significant SNPs co-localized with two GWAS results; the other category is SNP clusters (SCs) that reached a significant level in the GS-assisted GWAS. The preassembled *P. cathayana* genome in the laboratory was used as the reference genome, and genes within 20 kb upstream and downstream of significant SNPs were screened as candidate genes.

### Transcriptome sample collection and RNA-seq 

To investigate the pattern changes in xylem gene expression at two N levels and to explore potential N utilization-related genes, 13 growth promoting genotypes (A1, A2, A3, B2, C1, C2, C2, C3, D1, E2, E3) were selected from five natural populations (Lantian A, Yixian B, Longquan C, Fengning D, Youyu E). In mid-August, the stem bark at the breast diameter of the selected genotypes was peeled off, and the developing xylem was scraped from the xylem surface using a razor blade and immediately frozen in liquid N. Three biological replicates were performed for each genotype in the N-applied and non-N-applied blocks, respective. The Illumina NovaSeq™ 6000 high-throughput sequencing platform was used to perform RNA-seq on the library. After quality control of the offline data, *Populus trichocarpa* 3.1 was used as the reference genome (http://plants.ensembl.org), and Hisat2 software was used for sequence alignment analysis [57].

### Functional enrichment analysis of DEGs

To investigate the differences in response of different genotypes of *P. cathayana* to N and the regulatory mechanisms, we used fragments per kilobase million (FPKM) values to evaluate gene expression levels, and used fold change | log2FC |> 1 and significance level *p* < 0.05 as the screening standard for DEGs. Functional enrichment analysis of the DEGs were performed using Gene Ontology (GO) [58] and Kyoto Encyclopedia of Genes and Genomes (KEGG) [59] databases, with a significance level of *p* < 0.05.

### Gene co-expression network analysis

To further identify genes related to N utilization, we measured 8 traits related to N utilization as WGCNA associated traits, including PH, GD, aboveground biomass, leaf weight, xylem carbon content, xylem N content, bark carbon content, and bark N content. Aboveground biomass was determined after air drying, and leaf weight was the dry weight of each leaf. The C and N content was determined using an elemental analyzer after grinding the stem segments and bark. Before constructing the co-expression network, we removed genes with a total FPKM expression of less than 30 in all samples, and removed outliers by clustering analysis. The soft threshold function was used to calculate the weights, and then the modules were divided according to the dynamic hybrid shearing method, with a minimum of 150 genes per module. Modules with similar expression patterns (similarity = 0.75) were merged, and the merged modules were associated with traits using association analysis. Finally, Cytoscape 3.7.1 software was used to visualize the gene interaction network and screen hub genes.

### Identification of GWAS candidate genes and real-time fluorescence quantitative PCR validation

The combination of GWAS and WGCNA significantly enhances the capacity to identify core genes. To further identify the core genes in the GWAS candidate genes, we first homology-matched the *P. cathayana* genome with the reference genome and then overlapped the GWAS-identified candidate genes with the DEGs. Based on the transcriptome expression data, we evaluated the candidate gene expression patterns under fertilization conditions and further screened the candidate genes by combining gene annotation and enrichment results. To verify the reliability of RNA-Seq analysis and the expression levels of candidate genes, qRT-PCR analysis was performed with β-actin as an internal reference gene, and one genotype was randomly chosen in each of the five regions. Primers were designed using Primer Primer 6.0 software, and primer information for the four differentially expressed genes and the eight candidate genes were shown in Supplementary Table S11. Three technical replicates and three biological replicates were used for each genotype, and 2^−ΔΔCt^ was used to calculate the relative expression. The reaction system and reaction procedure refer to the description of TB Green® Premix Ex Taq™ II (TaKaRa, Dalian, China).

## Supplementary Information

**Additional file 1:** **Fig. S1.** Normality test of phenotypic data. (a) Normal distribution of PH in the N-applied area. (b) Normal distribution of GD in the N-applied area. (c) Normal distribution of PH in the no-N-applied area. (d) Normal distribution of GD in the no-N-applied area. **Fig. S2.** QQ plots of GWAS association analysis. (a) QQ plot of Ratio-PH GWAS correlation analysis. (b) QQ plot of GEBV-PH GWAS correlation analysis. (c) QQ plot of Ratio-GD GWAS correlation analysis. (d) QQ plot of GEBV-GD GWAS association analysis. **Fig. S3.** WGCNA quality control results. (a) Sample clustering tree; outlier samples C12 and D12 were eliminated. (b) Determination of the soft threshold; the soft threshold used in this study is β = 9. (c) Cluster tree and network heatmap, divided into 18 co-expression modules. **Fig. S4.** Greenyellow module gene interaction network and hub gene analysis. (a) Greenyellow module gene network visualization. (b) Hub gene function annotation and expression heatmap. Heatmap data were derived from normalized gene expression in each region population in RNA-seq. **Fig. S5.** Heatmap of DEG expression in N metabolism-related pathways. (a) Heatmap of carbon metabolism pathways. (b) Heatmap of N metabolism pathways. (c) Heatmap of the amino acid biosynthesis pathway. Heatmap data were derived from the normalized gene expression in each region population in RNA-seq. **Fig. S6.** The protein interaction network diagram of the N metabolism-related genes; the pink mark indicates the hub gene.

**Additional file 2:** **Supplementary Table S1.** GS anlysis of phenotypic data and GEBV values. **Supplementary Table S2.** GWAS analyzes statistical information. **Supplementary Table S3.** GWAS association loci and candidate gene information. **Supplementary Table S4.** Functional annotation information of GWAS candidate genes in the *P. cathayana* genome. **Supplementary Table S5.** KEGG enrichment results of GWAS candidate genes. **Supplementary Table S6.** The average Ratio-GD and Ratio-PH of the 34 regional Populus cathayana populations. **Supplementary Table S7.** Transcriptome sample quality control results. **Supplementary Table S8.** Gene ID and functional annotation of the pink module. **Supplementary Table S9.** gene ID and functional annotation of the greenyellow module. **Supplementary Table S10.** GWAS and transcriptome results overlap gene ID and expression level. **Supplementary Table S11.** qRT-RCR primer sequences.

## Data Availability

The data in this study will be published publicly. RNA-seq data have been uploaded to the Sequence Read Archive (https://www.ncbi.nlm.nih.gov/sra) under Bioproject PRJNA851202. Additional data and materials can be provided by the corresponding author on demand.

## Abbreviations

DEGs: Differentially expressed genes
GD: Ground diameter
GEBV: Genomic estimated breeding values
GS: Genomic selection
GWAS: Genome-wide association studies
MLM: Mixed linear model
N: Nitrogen
NUE: Nitrogen use efficiency
PCA: Principal component analysis
PH: Plant height
qRT‒PCR: Quantitative real-time fluorescence PCR
rrBLUP: Ridge regression best linear unbiased prediction
SNP: Single nucleotide polymorphism
TRN: Transcriptional regulatory network
WGCNA: Weighted gene co-expression network analysis
