# Supplementary material for: Combining GS-assisted GWAS and transcriptome analysis to mine candidate genes for nitrogen utilization efficiency in Populus cathayana
Source: BMC Plant Biol. 2023 Apr 5;23:182. doi: 10.1186/s12870-023-04202-1 (PMC10074878; doi:10.1186/s12870-023-04202-1)
Supplement: Supplementary file 1 — Additional file 1: Fig. S1. Normality test of phenotypic data. (a) Normal distribution of PH in the N-applied area. (b) Normal distribution of GD in the N-applied area. (c) Normal distribution of PH in the no-N-applied area. (d) Normal distribution of GD in the no-N-applied area. Fig. S2. QQ plots of GWAS association analysis. (a) QQ plot of Ratio-PH GWAS correlation analysis. (b) QQ plot of GEBV-PH GWAS correlation analysis. (c) QQ plot of Ratio-GD GWAS correlation analysis. (d) QQ plot of GEBV-GD GWAS association analysis. Fig. S3. WGCNA quality control results. (a) Sample clustering tree; outlier samples C12 and D12 were eliminated. (b) Determination of the soft threshold; the soft threshold used in this study is β = 9. (c) Cluster tree and network heatmap, divided into 18 co-expression modules. Fig. S4. Greenyellow module gene interaction network and hub gene analysis. (a) Greenyellow module gene network visualization. (b) Hub gene function annotation and expression heatmap. Heatmap data were derived from normalized gene expression in each region population in RNA-seq. Fig. S5. Heatmap of DEG expression in N metabolism-related pathways. (a) Heatmap of carbon metabolism pathways. (b) Heatmap of N metabolism pathways. (c) Heatmap of the amino acid biosynthesis pathway. Heatmap data were derived from the normalized gene expression in each region population in RNA-seq. Fig. S6. The protein interaction network diagram of the N metabolism-related genes; the pink mark indicates the hub gene. [file 12870_2023_4202_MOESM1_ESM.docx]

## **Supplementary figure**


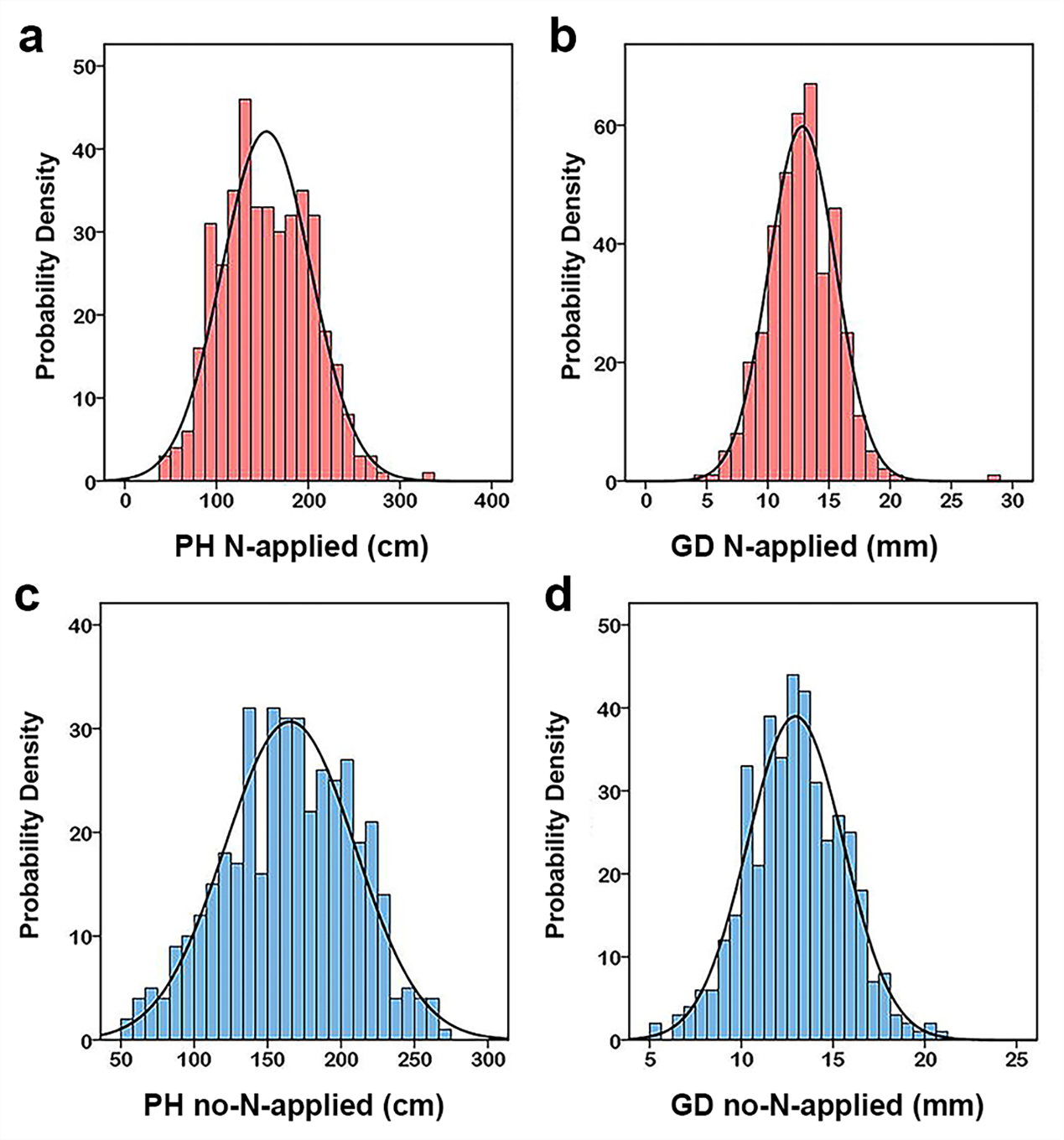


**Fig. S1** Normality test of phenotypic data. (a) Normal distribution of PH in the N-applied area. (b) Normal distribution of GD in the N-applied area. (c) Normal distribution of PH in the no-N-applied area. (d) Normal distribution of GD in the no-N-applied area.


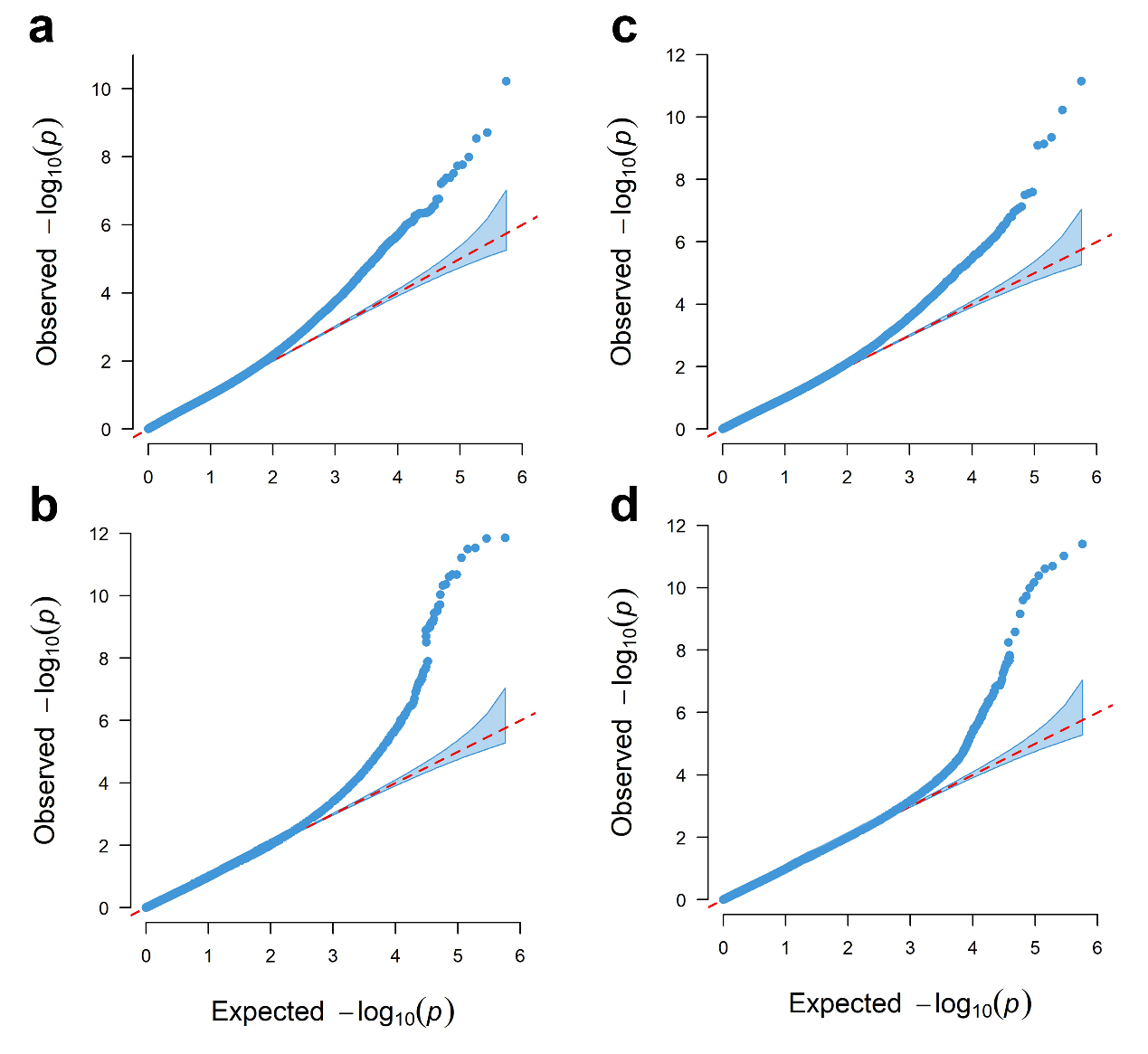


Fig. S2 QQ plots of GWAS association analysis. (a) QQ plot of Ratio-PH GWAS correlation analysis. (b) QQ plot of GEBV-PH GWAS correlation analysis. (c) QQ plot of Ratio-GD GWAS correlation analysis. (d) QQ plot of GEBV-GD GWAS association analysis.


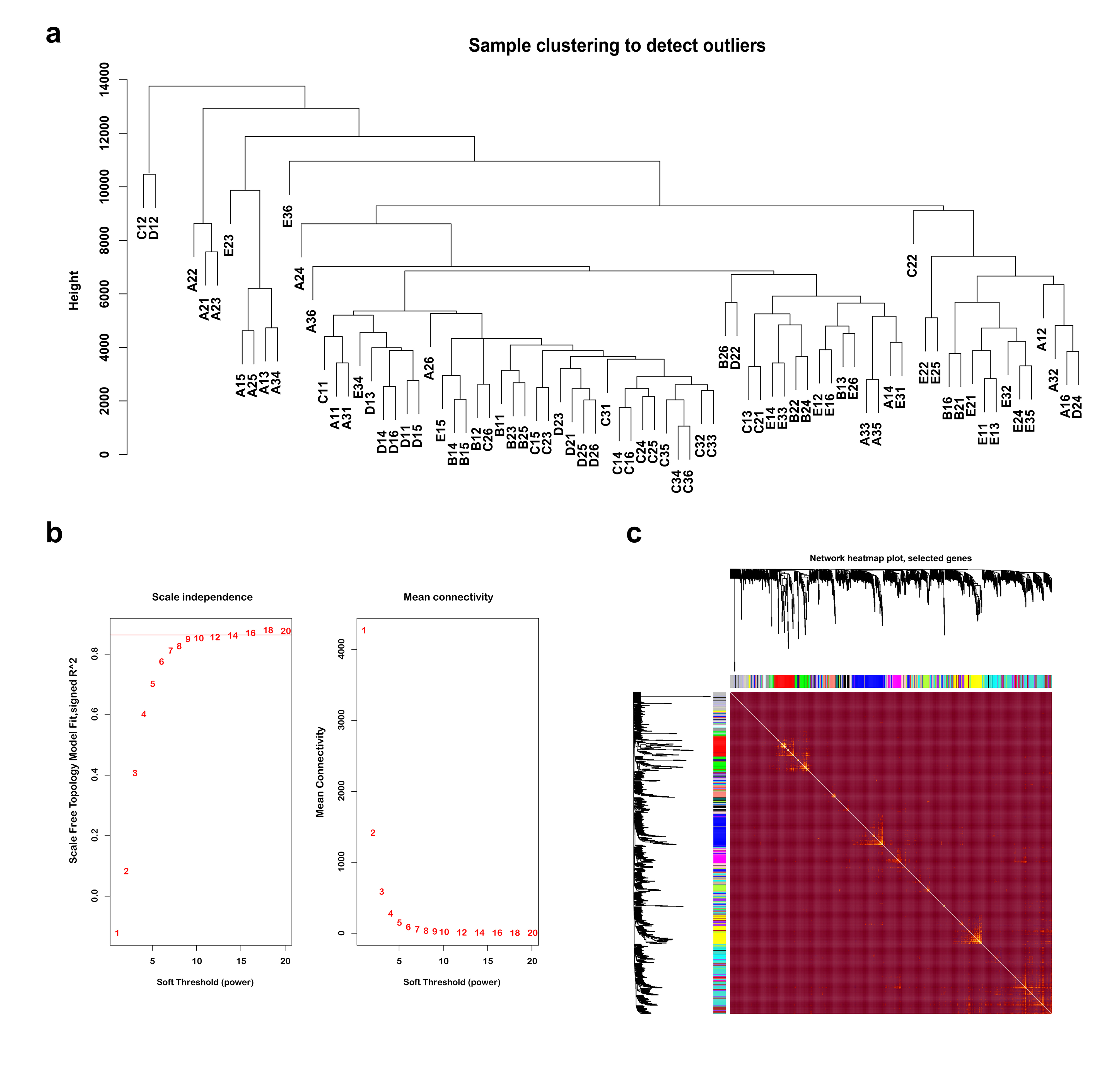


**Fig. S3** WGCNA quality control results. (a) Sample clustering tree; outlier samples C12 and D12 were eliminated. (b) Determination of the soft threshold; the soft threshold used in this study is β=9. (c) Cluster tree and network heatmap, divided into 18 co-expression modules.

**
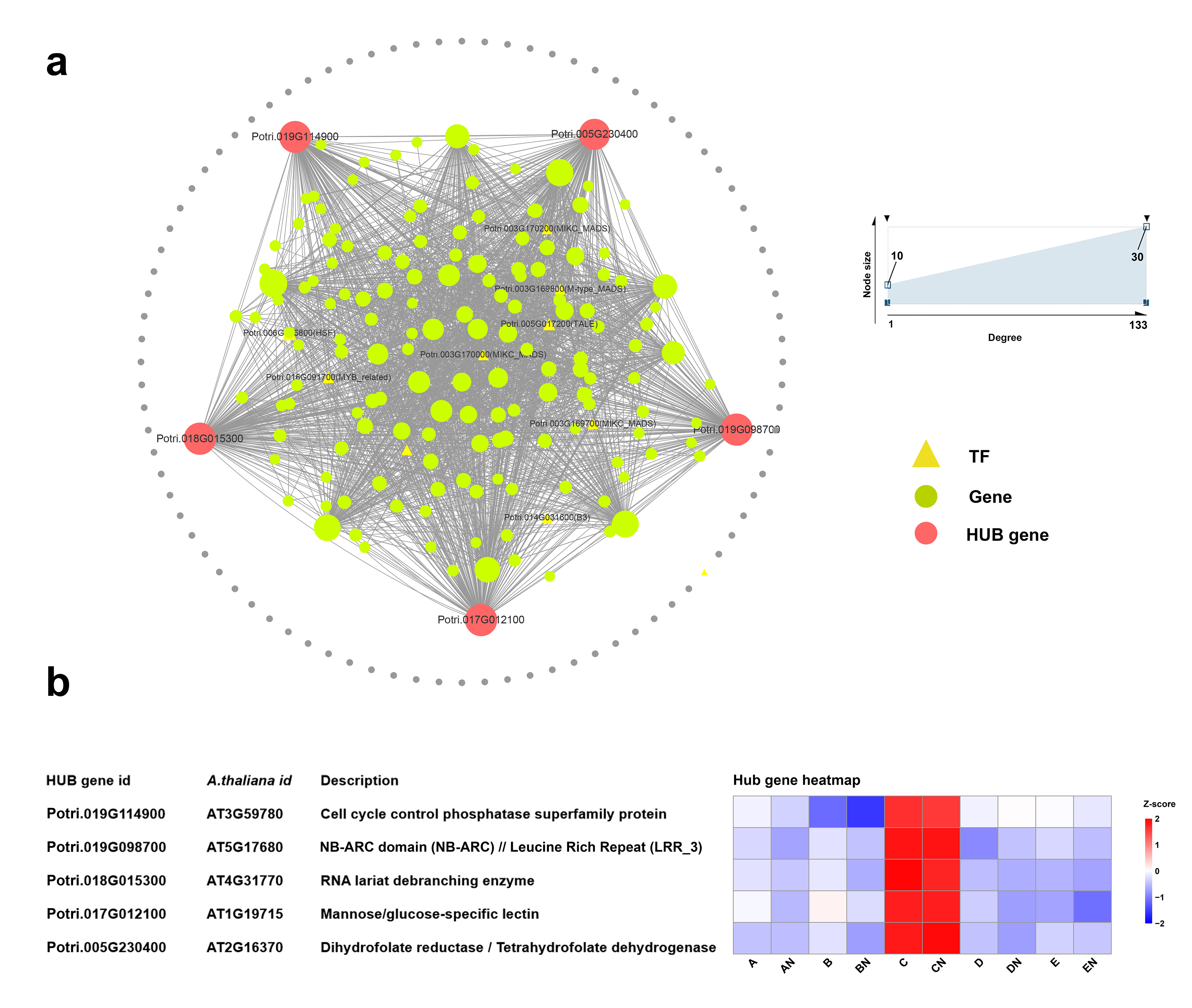
**

**Fig. S4** Greenyellow module gene interaction network and hub gene analysis. (a) Greenyellow module gene network visualization. (b) Hub gene function annotation and expression heatmap. Heatmap data were derived from normalized gene expression in each region population in RNA-seq.


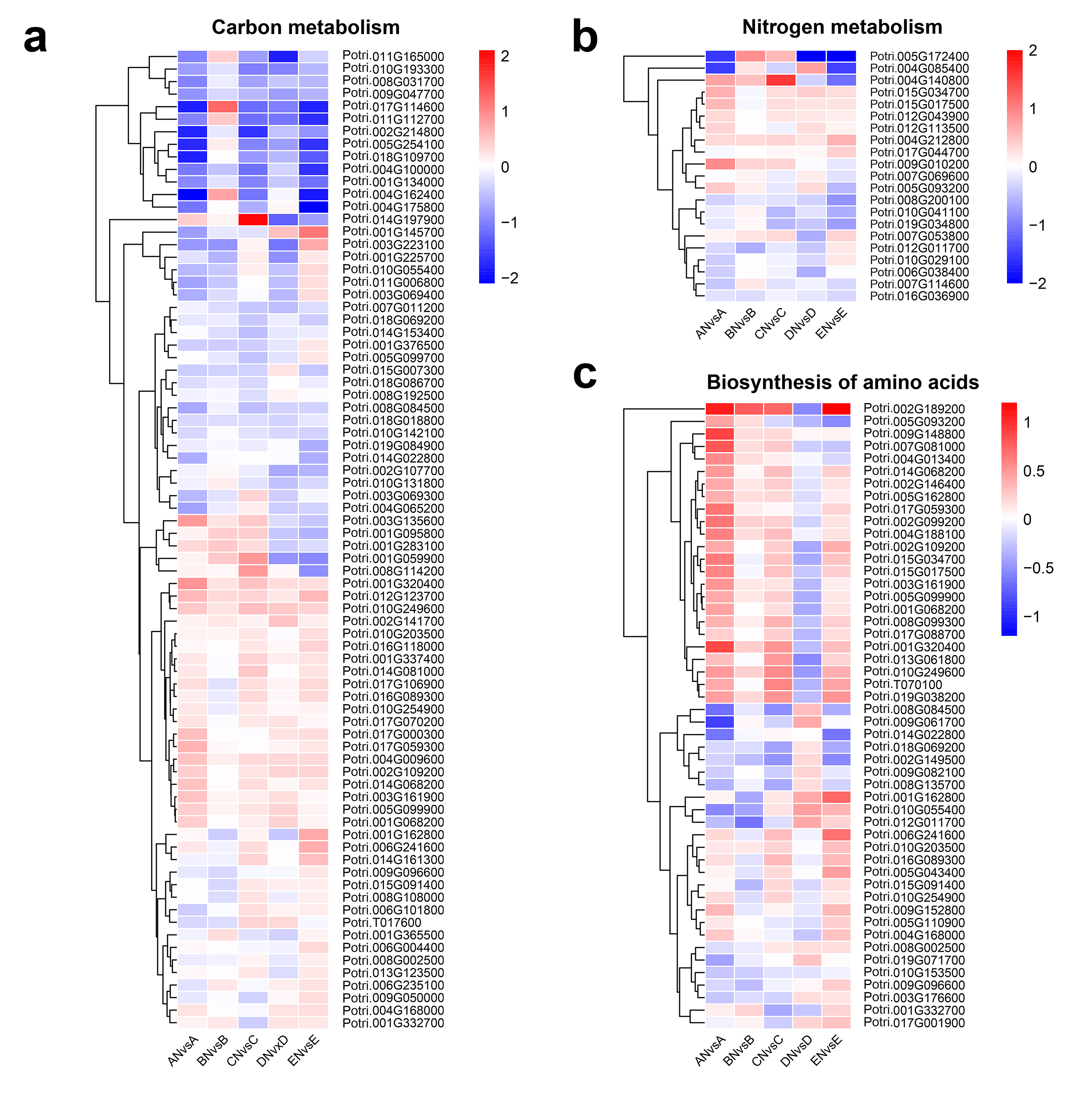


**Fig. S5** Heatmap of DEG expression in N metabolism-related pathways. (a) Heatmap of carbon metabolism pathways. (b) Heatmap of N metabolism pathways. (c) Heatmap of the amino acid biosynthesis pathway. Heatmap data were derived from the normalized gene expression in each region population in RNA-seq.


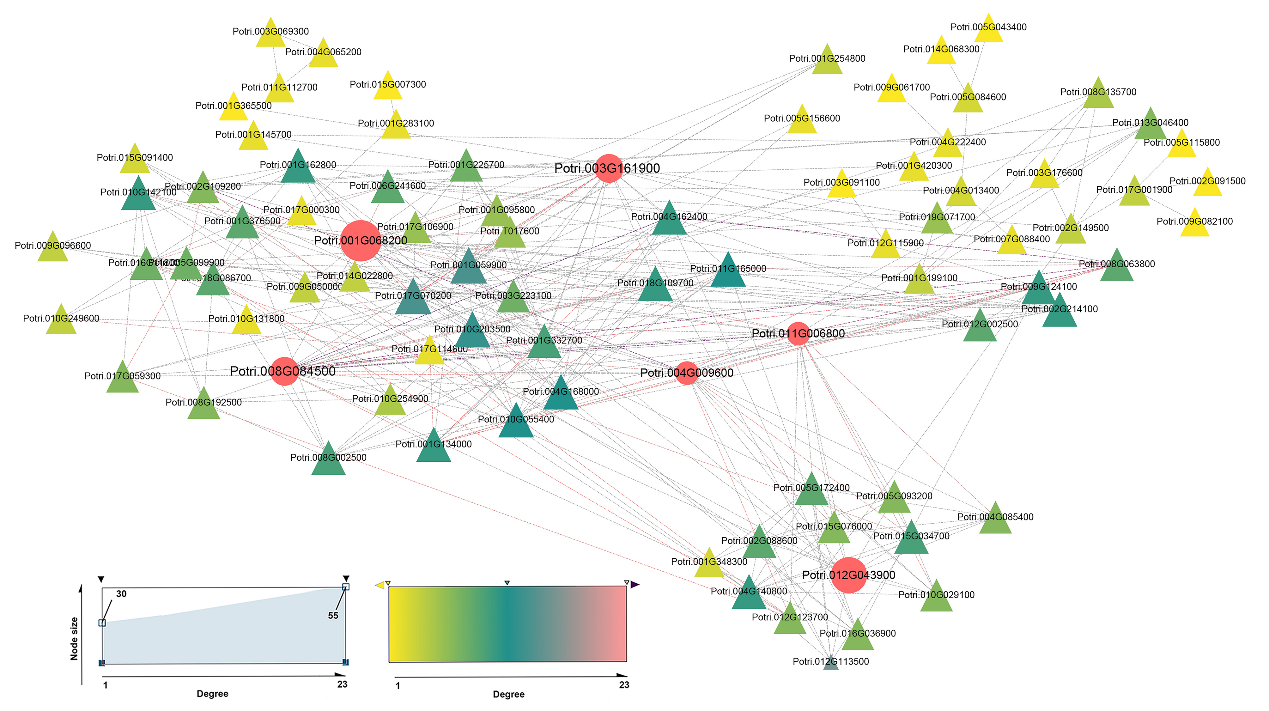


**Fig. S6** The protein interaction network diagram of the N metabolism-related genes; the pink mark indicates the hub gene.
